# An Iron–Complement Network Model of Thromboinflammation and Humoral Immune Remodeling in Severe COVID-19

**DOI:** 10.3390/cimb48050536

**Published:** 2026-05-21

**Authors:** Zhen Chen, Shanshan Wang, Yuzong Chen

**Affiliations:** 1 Institute of Drug Discovery Technology, Ningbo University, Ningbo 315211, China; 2311130004@nbu.edu.cn (Z.C.); chenyuzong@nbu.edu.cn (Y.C.); 2 Qian Xuesen Collaborative Research Center of Astrochemistry and Space Life Sciences, Ningbo University, Ningbo 315211, China

**Keywords:** complement activation, Thromboinflammation, iron metabolism, humoral immunity, severe COVID-19

## Abstract

Severe COVID-19 is characterized by profound thromboinflammatory and immune disturbances, but the network-level relationships among complement–coagulation dysregulation, humoral immune remodeling, and iron-associated immune regulation remain incompletely understood. Here, we performed integrative proteomic and transcriptomic analyses across peripheral blood and lung microenvironments using weighted gene co-expression network analysis (WGCNA), differential network analysis (DiNA), and immune deconvolution. Proteomic network analysis identified a disease-associated module enriched in complement activation, coagulation cascades, platelet degranulation, and acute inflammatory responses. Hub proteins, including C9, LBP, vWF, and F11, were prioritized based on module association and intramodular connectivity. Notably, C9 and LBP were repeatedly identified across WGCNA, DiNA, and differential expression analyses, underscoring their robust association with severe COVID-19-associated molecular network remodeling. Transcriptomic and CIBERSORTx-based immune deconvolution analyses showed altered immune-cell composition in blood and lung tissues, including B-cell and plasma-cell-associated changes. Notably, TFRC displayed cell-type-associated expression changes in naïve B cells and plasma cells, suggesting a potential link between iron-associated immune regulation and humoral immune remodeling. Collectively, these computational findings highlight coordinated complement–coagulation dysregulation, humoral immune remodeling, and TFRC-associated iron-related immune alterations in severe COVID-19, and prioritize TFRC, C9, and LBP as candidate molecular indicators requiring further experimental and clinical validation.

## 1. Introduction

Severe COVID-19 remains a major clinical challenge because a subset of patients develop rapidly progressive respiratory failure accompanied by systemic inflammation, coagulopathy, and multi-organ dysfunction [1]. Although mortality has declined since the acute phase of the pandemic, severe disease and its post-acute sequelae continue to impose a substantial health burden [2,3]. In particular, although complement activation, coagulation dysfunction, immune remodeling, and iron-related metabolic alterations have each been implicated in severe COVID-19 [4,5,6,7,8,9], how these processes are coordinated at the systems level remains incompletely understood. This unresolved relationship limits a comprehensive understanding of how thromboinflammatory and immune–metabolic disturbances jointly contribute to severe disease.

Severe COVID-19 is characterized by exaggerated inflammation and dysregulated immune responses [4,5]. Individuals with severe disease exhibit increased monocytes and neutrophils, decreased lymphocytes, and elevated levels of pro-inflammatory cytokines such as IL-6, IL-1β, tumor necrosis factor (TNF), MCP-1, and IP-10 [5,10]. Additionally, defects in type I interferon (IFN) activity have been observed [11,12]. These immune alterations are highly dynamic during the clinical course of the disease. Beyond cytokine-mediated inflammation, severe COVID-19 is also marked by endothelial injury, platelet activation, complement activation, and coagulation abnormalities [6,7,8,13,14]. These processes interact to form a thromboinflammatory state in which complement and coagulation pathways may mutually amplify vascular damage, immune activation, and disease progression [7,8,14,15,16]. Therefore, characterizing complement–coagulation dysregulation at the network level may provide insight into the molecular organization of severe disease.

Human cells function through complex interdependencies among molecular components. Diseases, including severe COVID-19, reflect disturbances in intricate intracellular and intercellular networks rather than individual genetic abnormalities [17]. Comprehending these network disruptions is essential for understanding their effects on tissue and organ systems and may help explain differences in disease risk and severity [18]. Because these processes involve coordinated interactions across multiple molecular and cellular layers, network-based and multi-omics approaches may provide important insights beyond single-gene or single-pathway analyses. WGCNA is well suited for identifying co-regulated molecular modules and hub proteins associated with disease traits [19,20], whereas DiNA can detect disease-associated changes in network topology and molecular connectivity that may not be captured by differential expression analysis alone [21]. Thus, combining WGCNA and DiNA allows both abundance-associated and connectivity-associated alterations to be examined in severe COVID-19.

In this study, we performed integrative proteomic and transcriptomic profiling of severe COVID-19 across lung tissue and peripheral blood microenvironments. We reasoned that network-based integration would identify complement–coagulation modules associated with thromboinflammation and reveal candidate molecules linking these proteomic changes with transcriptomic features of humoral immune remodeling and iron-associated immune regulation. To test this assumption, we applied WGCNA, DiNA, functional enrichment analysis, differential expression analysis, and CIBERSORTx-based immune deconvolution. This strategy was designed to prioritize network-associated molecules and generate testable hypotheses regarding coordinated thromboinflammatory and immune–metabolic dysregulation in severe COVID-19.

## 2. Materials and Methods

### 2.1. Data Collection

The serum protein expression dataset, including 17 severe COVID-19 patients and 18 healthy controls, was obtained from the publicly available serum proteomic dataset reported by Shen et al. [22]. RNA-seq data were sourced from two GEO datasets: GSE171110, which sequenced 54 blood samples (10 healthy controls, 44 severe COVID-19 patients), and GSE183533, which sequenced postmortem lung tissues from 31 severe COVID-19 patients and 10 control lung samples from non-infected cancer patients.

### 2.2. Cross-Dataset Integration Strategy

Because the datasets represented different molecular platforms and biological compartments, including serum proteomics, whole-blood transcriptomics, and postmortem lung transcriptomics, they were analyzed separately rather than directly merged into a single expression matrix. Cross-dataset integration was performed at the level of pathway enrichment, network features, immune-cell inference, and candidate molecule convergence.

### 2.3. Data Imputation and Outlier Removal

Handling missing data is a common challenge, as removing incomplete entries can reduce valuable information and limit meaningful inferences [23]. To address this, we filtered out samples and genes with over 50% missing data or zero variance. Missing values were then imputed using the missForest method, a non-parametric approach based on random forests [24]. Additionally, hierarchical clustering with complete linkage and Euclidean distance was used to identify and remove outlier samples.

### 2.4. Protein Co-Expression Network Construction

We used WGCNA [19] to construct a weighted protein co-expression network based on serum proteomic data. Pearson correlation coefficients were calculated between protein pairs to create a similarity matrix, which was then converted into an adjacency matrix. A topological overlap matrix (TOM) was generated to reduce noise, and hierarchical clustering based on dissimilarity (1-TOM) was applied to define co-expression modules. Modules were merged using Module Eigengene (ME) to summarize protein abundance profiles. Network construction and module detection were performed using the blockwiseModules function in the WGCNA package [20] with key parameters: power = 3, networkType = unsigned, TOMType = signed, minModuleSize = 30, mergeCutHeight = 0.25.

The soft-thresholding power was selected based on the scale-free topology fit index and mean connectivity (Supplementary Figure S1). A power of 3 was chosen because it was the first value at which the scale-free topology fit index reached 0.85 and showed an approximately scale-free connectivity distribution at β = 3 (R^2^ = 0.93, slope = −1.77), while retaining adequate mean connectivity.

### 2.5. Identification of Co-Expressed Protein Modules and Hub Proteins

We identified co-expressed protein modules associated with severe COVID-19 by correlating Module Eigengenes (ME) with clinical traits using Pearson correlation, visualized through the labeledHeatmap function in WGCNA. The module with the strongest correlation to severe COVID-19 was selected for further analysis. Gene Significance (GS, used here as protein-trait significance) quantified the correlation between protein abundance and clinical traits, while Module Membership (MM) measured the correlation between protein abundance and ME. Hub proteins were defined as those with |MM| > 0.8 and |GS| > 0.8. These thresholds were used to prioritize high-confidence hub proteins with both strong module membership and strong disease association. Proteins meeting these criteria were further ranked by intramodular connectivity (kWithin) to identify topologically central nodes within the disease-associated module.

### 2.6. Differential Network Analysis

To examine changes in module topology between healthy (reference set) and severe COVID-19 (test set) groups, we used the modulePreservation function in WGCNA. We assessed module preservation using Z*_summary_* and medianRank statistics [21], Z*_summary_* indicates the significance of observed data, calculated as the average of Z-scores from density and connectivity measures. A Z*_summary_* < 2 suggests no preservation, 2–10 indicates weak to moderate preservation, and > 10 indicates strong preservation. However, Z*_summary_* is influenced by module size, while medianRank is less size-dependent and allows for relative preservation comparisons. Modules with lower medianRank values are more preserved than those with higher values. In this study, a module was defined as non-preserved (differential network) if Z*_summary_* ≤ 2 and medianRank was highest.

For protein *i*, overall network connectivity in the weighted networks of the test set and reference set is denoted as k_1_(i) and k_2_(i), respectively. To account for network size, connectivity for each protein is divided by the maximum network connectivity, as shown in Equations (1) and (2).

(1) $$K_{1}(i)=\frac{k_{1}(i)}{max(k_{1})}$$

(2) $$K_{2}\left(i\right)=\frac{k_{2}\left(i\right)}{\max\left(k_{2}\right)}$$

Subsequently, we calculated the differential connectivity of protein *i* within the identified differential network module according to Equation (3). The top 20 proteins ranked in descending order by differential connectivity were selected as hub proteins. In this analysis, reduced module preservation or high differential connectivity was interpreted as indicative of disease-associated network rewiring. Differential connectivity indicates that the correlation structure or network neighborhood of a protein differs between healthy and severe COVID-19 conditions, suggesting altered molecular co-regulation.

(3) $$DiffK(i)=|K_{1}(i)-K_{2}(i)|$$

### 2.7. Functional Enrichment Analysis

We used Metascape [25] for biological functional enrichment analysis of proteins in modules positively correlated with severe COVID-19. The analysis included the following ontology sources, GO Biological Processes, KEGG Pathway, Reactome Gene Sets, DisGeNET, and WikiPathways, using all human genome genes as the background. The Benjamini–Hochberg (BH) method [26] was applied to adjust *p*-values for multiple testing, and entries with an adjusted *p*-value < 0.01 and a minimum count of 3 were collected for clustering based on similarity.

### 2.8. Differential Expression Analysis

We employed the limma [27] model for differential expression analysis between severe COVID-19 patients and healthy controls. Log2 fold-change (log2FC) values were calculated, and statistical significance was evaluated using empirical Bayes moderated statistics. The BH method was applied to control the false discovery rate (FDR). Proteins with FDR < 0.05 and |log2FC| > 0.26 were considered differentially expressed. The |log2FC| threshold of 0.26 corresponds to approximately a 1.2-fold change and is close to the |log2FC| > 0.25 effect-size cutoff used in previous serum proteomic analyses of COVID-19 [22].

### 2.9. Impute Immune Cell Fractions with CIBERSORTx

We employed CIBERSORTx [28] to impute immune cell fractions and gene expression patterns without physical cell isolation. Raw gene expression profiles (GEPs) from bulk RNA-seq were preprocessed to transcript-per-million (TPM) without nonlinear log2 transformation or negative/missing values. We employed the LM22 [29] signature matrix to profile 22 human immune cell types, including B cells, T cells, and macrophages. B-mode batch correction was applied for cross-platform deconvolution, with 1000 permutations employed to assess performance. Samples with empirical *p*-values < 0.05 were included in further analyses. The Wilcoxon rank-sum test evaluated differences in immune cell subtype distributions between severe COVID-19 patients and healthy controls, with FDR < 0.05 set as the significance threshold.

Because CIBERSORTx infers immune-cell proportions from bulk RNA-seq profiles, the estimated cell fractions may be affected by inflammation-associated transcriptional changes, tissue heterogeneity, RNA quality, and reference signature mismatch, particularly in postmortem lung tissues. Therefore, the inferred immune-cell fractions were interpreted as relative estimates of immune composition rather than direct measurements of cell abundance.

### 2.10. Impute Cell-Type-Specific Gene Expression

We used the group-mode of CIBERSORTx to impute cell type-specific transcriptional differences between severe COVID-19 patients and healthy controls. Employing adaptive noise-filtered GEPs, we imputed statistically significant differentially expressed genes for a specific immune cell type using an R script. The cut-off criteria were set at FDR < 0.05.

### 2.11. Statistical Analyses

All data preprocessing and statistical analyses were performed in R software (version 3.6.1).

## 3. Results

### 3.1. Severe COVID-19 Linked to Complement Dysregulation and Thromboinflammation Signs

**Figure 1:**
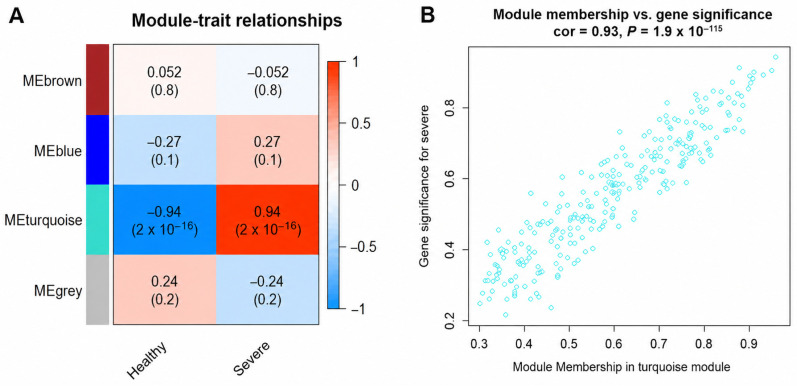
Correlation between module and clinical phenotype. (**A**) Module-trait relationships. Each row corresponds to a module eigengene, the column to a trait. Each cell contained the corresponding correlation and *p*-value. High correlations were colored in red, low correlations in blue. (**B**) A scatterplot of gene significance versus module membership in the turquoise module.

Hierarchical clustering based on TOM dissimilarity revealed three co-expression modules (Supplementary Figure S2A). The heatmap depicts the topological overlap dissimilarity among these modules (Supplementary Figure S2B). To identify significant modules associated with severe COVID-19, we analyzed ME and module significance (MS). Among the three modules (blue, brown, turquoise), the turquoise module, comprising 263 proteins, exhibited a strong positive correlation with severe COVID-19 (r = 0.94, *p* = 2 × 10^−16^; Figure 1A). The robustness of this correlation, given the moderate sample size, is further evaluated through module preservation statistics and cross-validation in the differential network analysis below. Detailed information on the co-expressed proteins is provided in Supplementary Table S1. As shown in Figure 1B, there is a significant correlation between GS and MM within the turquoise module, indicating that hub proteins are closely associated with the COVID-19 phenotype. Subsequent analyses focused specifically on this module.

**Figure 2:**
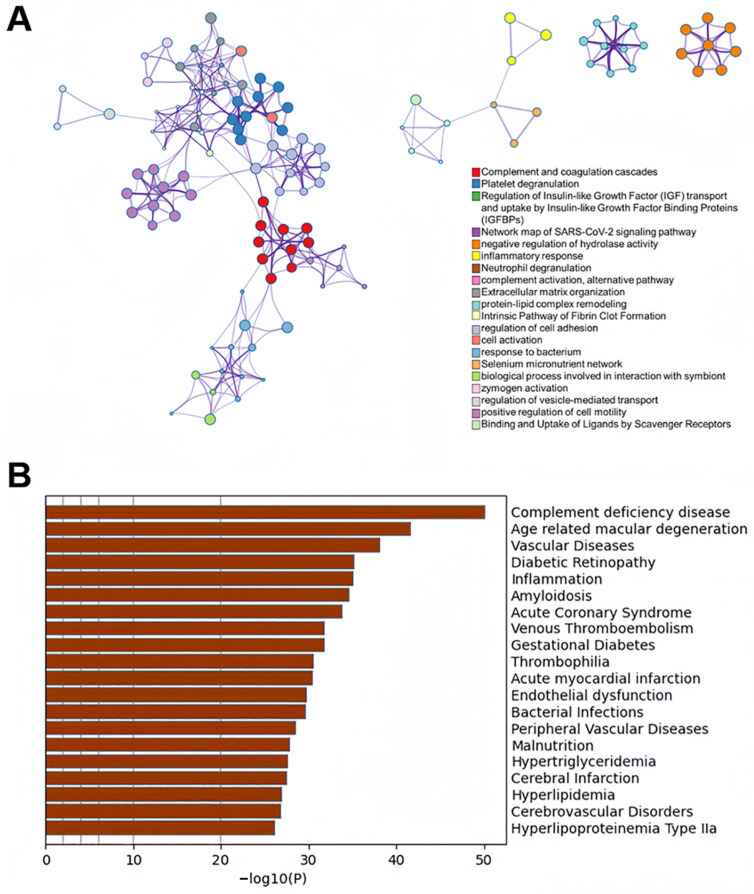
Gene enrichment analysis and network visualization. (**A**) Pathway enrichment analysis: each term is represented by a circle node, with node size proportional to the number of input genes associated with the term, and color representing cluster identity. Terms with a similarity > 0.3 are connected by edges. (**B**) Summary of gene–disease association analysis.

To investigate the biological functions of proteins in the turquoise module, we performed a functional enrichment analysis of the encoding genes. After eliminating redundant pathways, the top 20 significant biological processes are presented in network format (Figure 2A, Supplementary Table S2). The results indicated significant enrichment of co-expressed genes in pathways related to host immune response, inflammatory response, and coagulation. Notably, proteins involved in the complement and coagulation cascades, as well as platelet degranulation, exhibited severe dysregulation. While complement activation in COVID-19 is well-documented [6,13], our network analysis reveals that complement and coagulation proteins do not merely co-exist but are topologically integrated within a single co-expression module. The tight correlation of this module with disease severity (r = 0.94) suggests that thromboinflammation operates as a coordinated systems-level program rather than isolated pathway activation. Notably, platelet degranulation genes were embedded within this same module, indicating that platelet-mediated signaling may serve as a structural bridge between complement and coagulation dysregulation in severe disease.

DisGeNET [30] is a comprehensive database integrating information on genes and mutations related to human diseases. To explore the relationship between proteins in the turquoise module and diseases, we conducted gene–disease association analysis using Metascape (Figure 2B). The proteins in this module are significantly associated with conditions such as complement deficiency, vascular diseases, thromboembolism, amyloidosis, endothelial dysfunction, diabetes, and cerebrovascular disorders. These findings highlight the proteins’ critical role in multi-organ failure caused by acute SARS-CoV-2 infection, involving inflammatory responses, endothelial damage, and thrombotic events. Further investigation of these proteins will enhance our understanding of COVID-19 pathogenesis and identify potential therapeutic targets.

We identified proteins in the turquoise module as hub proteins based on the criteria |MM| > 0.8 and |GS| > 0.8, ranking their significance by network topological attribute kWithin (Table 1). Among the 20 hub proteins, 19 were significantly differentially expressed in severe COVID-19 patients compared to healthy controls, with 14 upregulated and 5 downregulated (Supplementary Figure S3).

Notably, upregulated proteins such as SERPING1, C2, C6, C9, F11, vWF, and C4BPA are involved in the complement and coagulation cascade pathways, which significantly impact the severity of SARS-CoV-2 infection. SERPING1 encodes the C1 esterase inhibitor (C1-INH), a key regulator of complement activation [14]. C6 and C9 are components of the complement membrane attack complex (MAC), crucial for mediating cell damage [31]. These findings support anti-inflammatory strategies targeting the complement system.

Consistent with prior reports of endothelial damage in COVID-19 [7], vWF was significantly elevated in our severe cohort, and its high intramodular connectivity (kWithin = 33.34) positioned it as a structural hub within the thromboinflammatory module. Similarly, F11’s hub status in our network analysis aligns with evidence that the contact activation system may contribute to COVID-19-associated thromboinflammation [16]. Although heparin-based anticoagulation has been associated with reduced mortality in severe COVID-19 patients with coagulopathy [32], targeted modulation of contact activation remains a potential strategy requiring further investigation. Monitoring serum levels of vWF and F11 after SARS-CoV-2 infection may inform therapeutic strategies to manage COVID-19 severity and associated thrombotic complications.

A recent multicenter longitudinal study by Cervia-Hasler et al. involving 113 patients who either fully recovered from COVID-19 or developed Long COVID found that local activation of the innate immune complement system may contribute to thrombo-inflammation and hinder recovery [33,34]. Notably, patients with Long COVID showed significant upregulation of serum vWF and F11. Thus, our findings not only provide a basis for new diagnostic solutions but also suggest potential interventions for treating thrombo-inflammation associated with Long COVID.

### 3.2. Network Topology Alterations in Key Proteins Uncover Humoral Immune Dysregulation in Severe COVID-19

Co-expression networks were constructed for healthy controls and severe COVID-19 patients (Supplementary Figure S4). This healthy network was then mapped onto the severe COVID-19 cohort, and DiNA was performed to identify non-conserved modules related to survival and disease progression.

**Figure 3:**
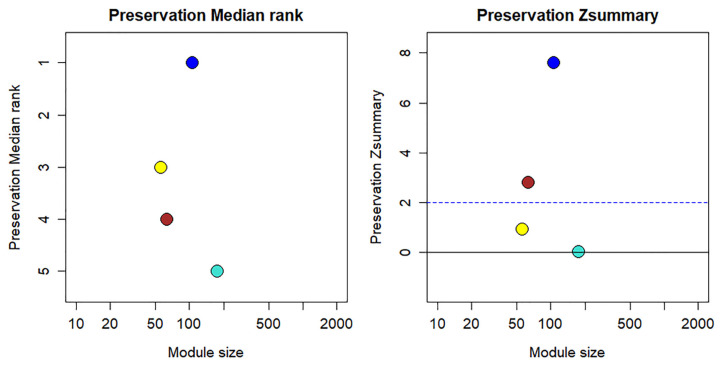
Composite preservation statistics Z*_summary_* and medianRank taking healthy controls as reference and severe COVID-19 as test networks. Each point represents a module, labeled by color. The dashed blue line indicates the thresholds Z*_summary_* = 2.

The turquoise module exhibited the lowest Z*_summary_* score and the highest medianRank among all modules (Figure 3), meeting the formal criteria for a differential network (non-preserved module). This indicates that SARS-CoV-2 infection induces not merely quantitative expression changes but qualitative rewiring of protein-protein association patterns—specifically, the complement–coagulation hub proteins reorganize their network connectivity in severe disease. The topological plasticity of this module suggests that therapeutic targeting of complement–coagulation components may have context-dependent efficacy depending on disease stage.

**Figure 4:**
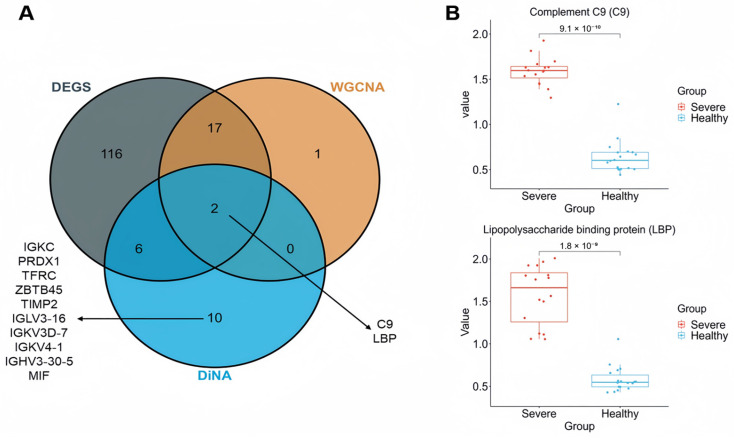
Hub Protein Overlap and Differential Gene Expression in Severe COVID-19 Patients. (**A**) Overlap of hub proteins identified by DiNA, WGCNA modules, and differentially expressed genes (DEGs) between severe COVID-19 patients and healthy controls. (**B**) Comparative expression levels of C9 and LBP in severe COVID-19 patients vs. healthy controls.

To identify hub proteins in the non-conservative module, we calculated the absolute value of the weighted connectivity change (DiffK) for each protein across the reference and test networks. The top 20 proteins with the highest DiffK values were prioritized as hub proteins (Supplementary Table S3). Functional enrichment analysis revealed that these hub proteins are primarily associated with pathways related to the adaptive immune response, immune effector processes, acute-phase response, SARS-CoV-2 signaling, and platelet degranulation (Supplementary Figure S5). Notably, C9 and LBP function as hub proteins in both the non-conservative module and the module linked to severe COVID-19 (Figure 4A), and they show differential expression between severe COVID-19 patients and healthy controls (Figure 4B).

In contrast to the confirmatory findings for vWF and F11, C9 and LBP represent a novel systems-level observation of this study. While C9 (terminal complement pathway) and LBP (innate immune sensing) have been individually implicated in complement activation and LPS response [31,35,36,37,38], their co-identification as differential network hubs (DiffK-ranked top 20) and their convergence in both WGCNA and DiNA analyses have not been previously reported. Specifically, the topological reorganization of C9 and LBP within the same non-preserved module suggests a mechanistic coupling between membrane attack complex formation and endotoxin signaling in severe disease. Both proteins showed significant differential expression between severe COVID-19 patients and healthy controls (Figure 4B). Therefore, measuring serum levels of C9 and LBP in individuals testing positive for SARS-CoV-2 may serve as a valuable diagnostic tool for identifying patients at risk for severe outcomes.

Within the non-conservative module, ten proteins (IGKC, PRDX1, TFRC, ZBTB45, TIMP2, IGLV3-16, IGKV3D-7, IGKV4-1, IGHV3-30-5, MIF) exhibited significant changes in network topology in severe COVID-19 patients compared to healthy controls, despite no notable differences in serum expression levels. This finding highlights alterations in the host protein interaction landscape post-SARS-CoV-2 infection that conventional differential expression analyses may overlook.

Functional enrichment analysis revealed significant involvement of these proteins in pathways related to immunoglobulin production (GO:0002377) and positive regulation of B cell activation (GO:0050871), indicating their roles in antibody production and B cell activation, which may reflect the humoral immune characteristics in severe COVID-19.

To further investigate these proteins’ roles in the immune system, we analyzed transcriptomic data from blood and lung samples of severe COVID-19 patients and healthy controls using CIBERSORTx. This analysis aims to determine the relative changes in humoral immune cell types and explore potential associations between these proteins and specific immune cell types in severe COVID-19. Insights from this work will enhance our understanding of the immunological mechanisms underlying severe COVID-19.

### 3.3. Immune Microenvironment in Severe COVID-19: Blood and Lung Tissues

**Figure 5:**
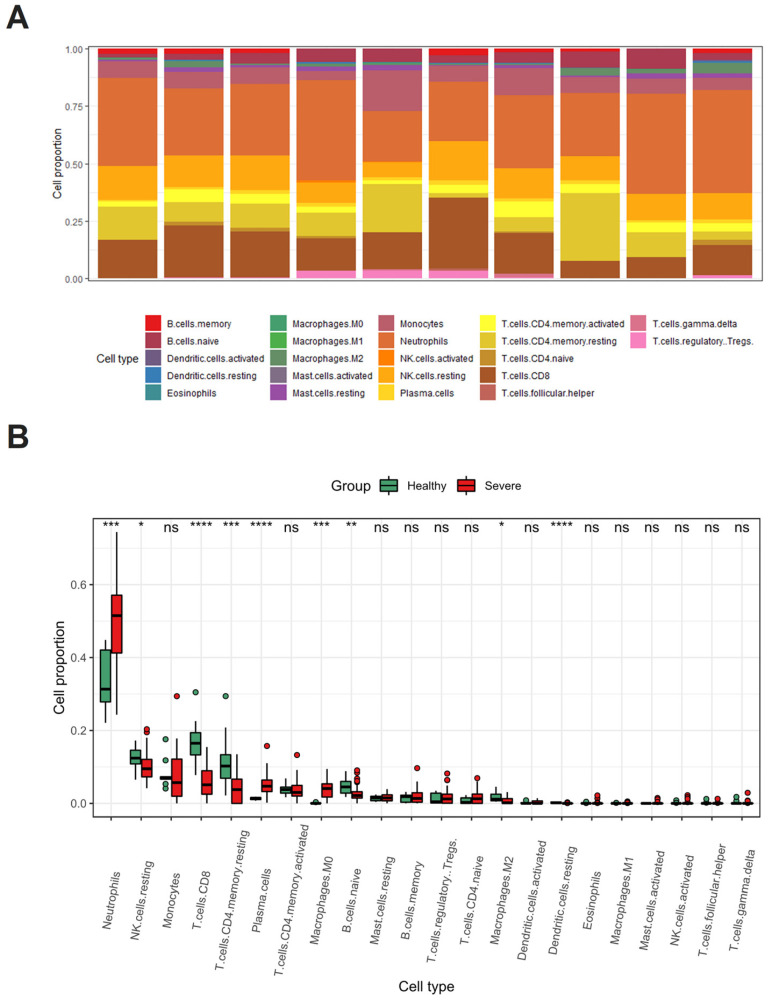
Immune cell subset proportions and distribution in healthy and severe COVID-19 blood samples. (**A**) Proportion of 22 immune cell subsets in healthy blood samples. (**B**) Differential distribution of selected immune cell subsets between severe COVID-19 and healthy controls (*p*-values from Wilcoxon test). ns, not significant; * *p* < 0.05; ** *p* < 0.01; *** *p* < 0.001; **** *p* < 0.0001.

Using CIBERSORTx, we calculated the infiltration scores of 22 immune cell types in both blood and lung tissues from severe COVID-19 patients and healthy controls. In healthy adults, the predominant immune cell types in the blood are neutrophils and lymphocytes (Figure 5A). In severe COVID-19 patients, neutrophil levels significantly increase (*p* < 0.001, Figure 5B), contributing to an excessive inflammatory response. Conversely, there is marked suppression of the adaptive immune response, evidenced by reduced proportions of specific lymphocyte populations, including CD8+ T cells, memory CD4+ T cells, and natural killer cells. As our study indicates, the activation of the coagulation system in COVID-19 patients leads to elevated production of pro-inflammatory cytokines and chemokines, resulting in the recruitment of lymphocytes and monocytes/macrophages to the lungs. This dysregulation subsequently causes damage to pulmonary endothelial and epithelial cells [39].

In terms of B cell dynamics, patients with severe COVID-19 show a significant upregulation of plasma cells, while naive B cells are markedly downregulated. This shift suggests a robust yet potentially dysregulated immune response. The increase in plasma cells indicates heightened antibody production aimed at neutralizing the virus, whereas the decrease in naïve B cells may reflect immune resource depletion and a risk of immune exhaustion. This imbalance could impair the body’s ability to effectively respond to the virus and may contribute to severe complications, such as hyperinflammation driven by excessive antibody production and immune complex formation [40].

Consistent with previous findings, the immune cell composition in healthy lung tissues predominantly consists of macrophages and lymphocytes (Supplementary Figure S6A). Wilcoxon rank-sum tests revealed significant increases in M1 macrophages, naïve B cells, and plasma cells in severe COVID-19 patients compared to healthy controls (Supplementary Figure S6B). The rise in M1 polarized macrophages is associated with acute inflammation and lung damage [41].

Notably, plasma cells were significantly upregulated in both blood and lung tissues of severe patients, suggesting a strong link between plasma cell proliferation and disease severity. The increase in naïve B cells in lung tissues, despite their decrease in blood, indicates a localized immune response driven by inflammation, likely recruiting new B cells to the infection site. The activation of the coagulation system and the production of pro-inflammatory cytokines and chemokines promote the recruitment of lymphocytes and monocytes/macrophages to the lungs, contributing to an inflammatory environment that disrupts the release of effective cytotoxic mediators, leading to further damage to pulmonary endothelial and epithelial cells [39].

These immune cell composition changes—specifically the expansion of plasma cells and depletion of naive B cells in blood, alongside M1 macrophage accumulation in lung tissue—are correlational findings consistent with dysregulated humoral and innate immune responses in severe COVID-19. While the cross-sectional design and computational deconvolution preclude causal inference, these patterns generate the testable hypothesis that therapeutic strategies balancing immune activation with inflammation control may support immune reconstitution during recovery.

### 3.4. Identification of Cell Type-Specific Features

**Figure 6:**
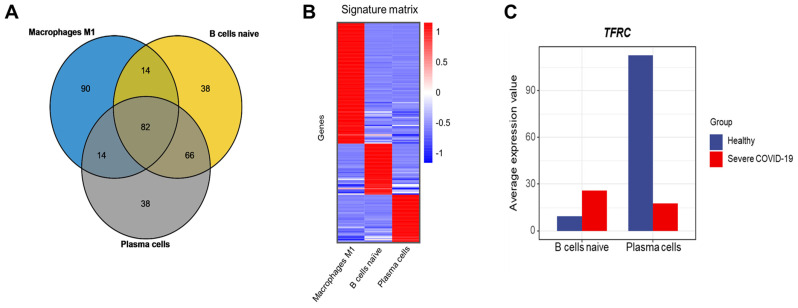
Cell type-specific features in immune cells. (**A**) Venn diagram of the top 200 genes from three specific immune cells extracted from the LM22 immune cell gene expression signature. (**B**) Signature matrix of three immune cell types. (**C**) TFRC gene expression across different immune cell types in healthy and severe COVID-19 patients.

We performed gene expression analysis on naïve B cells, plasma cells, and M1 macrophages from blood samples of severe COVID-19 patients and healthy controls. We identified the top 200 enriched genes for each cell type using the LM22 document. Venn diagram analysis was then used to examine the overlap and unique genes among these cell types (Figure 6A). Genes found in the non-overlapping regions were used to construct the signature matrix for each immune cell type. (Figure 6B).

Notably, TFRC was significantly upregulated in naive B cells of COVID-19 patients (adjusted *p*-value = 7.15 × 10^−6^) and downregulated in plasma cells (adjusted *p*-value = 2.16 × 10^−6^, Figure 6C). TFRC’s reciprocal expression pattern—upregulated in naive B cells and downregulated in plasma cells—suggests a stage-specific iron dependency during B-cell differentiation, though causality cannot be inferred from this cross-sectional analysis. We hypothesize that high TFRC in naive B cells supports the metabolic burst required for antigen-driven activation [9], whereas its downregulation in plasma cells may reflect a shift to antibody-secreting specialization with altered iron requirements. This pattern is compatible with ferroptosis susceptibility dynamics: excessive iron accumulation via TFRC can propagate lipid peroxidation and cell death [42,43], potentially contributing to the observed naive B-cell depletion in peripheral blood. Direct evidence for ferroptosis in this context, however, requires experimental validation beyond transcriptomic inference. These findings link TFRC-associated iron metabolism to humoral immune remodeling in severe COVID-19.

## 4. Discussion

In this study, we employed an integrative network-based framework combining WGCNA, DiNA, and immune deconvolution to investigate severe COVID-19. Unlike conventional differential expression analysis, which evaluates molecules largely as independent features, our approach reveals how proteins reorganize their co-expression relationships in disease states. This systems-level perspective uniquely identified two key phenomena: (i) the topological integration of complement and coagulation cascades into a single co-expression module, suggesting that thromboinflammation operates as a coordinated network program rather than isolated pathway activation; and (ii) the qualitative rewiring of this module in severe COVID-19, evidenced by its non-preservation between healthy and disease networks. These structural insights—particularly the convergence of C9 and LBP as differential network hubs—would not emerge from single-gene or single-pathway analyses.

The immune microenvironment is vital for both disease diagnosis and the efficacy of clinical immunotherapy. Our immune infiltration analysis revealed regulatory mechanisms of various immune cell populations in the lung tissue and peripheral blood of severe COVID-19 patients, contributing to excessive inflammation and lung damage. In recovered patients, the absence of naïve T cells and disrupted B cell maturation [44] indicate lasting effects on the adaptive immunity. The upregulation of plasma cells in both blood and lung tissues in severe cases reflects an aggressive antibody response, while the localized increase in naïve B cells in the lungs points to a targeted immune reaction at the infection site. These findings may contribute to long-term immune-related complications in COVID-19 survivors [45].

Significant alterations in iron metabolism were also observed, particularly regarding TFRC, which was upregulated in naïve B cells and downregulated in plasma cells in the blood of these patients. The association of TFRC with ferroptosis [46], as well as the role of ferroptosis in various conditions such as tissue injury, inflammation, neurodegenerative diseases, and cancer [47], underscores the critical importance of iron metabolism in COVID-19 pathogenesis. Although TFRC has been linked to ferroptosis in other biological contexts, ferroptosis markers such as lipid peroxidation, GPX4 activity, or direct iron measurements were not assessed in this study. Therefore, the potential relationship between TFRC dysregulation and ferroptosis in severe COVID-19 should be considered a mechanistic hypothesis requiring experimental validation rather than a conclusion directly supported by the present data.

The convergence of complement–coagulation dysregulation with TFRC-associated iron-related immune alterations is consistent with a hypothesized iron–complement network relationship in severe COVID-19. We propose a testable mechanistic hypothesis in which: (i) complement activation, reflected in part by C9 upregulation and MAC-related features, may contribute to endothelial injury, platelet-associated inflammatory responses, and a pro-thrombotic microenvironment; (ii) LBP-associated inflammatory signaling may reflect or contribute to systemic innate immune activation and immune-cell recruitment; (iii) altered TFRC expression in naïve B cells may reflect increased iron demand during lymphocyte activation and may also indicate vulnerability to iron-associated oxidative stress under severe inflammatory conditions; and (iv) plasma-cell expansion and naïve B-cell depletion may indicate humoral immune remodeling during severe disease. This framework draws on prior evidence that inflammatory cytokines such as IL-6 can induce hepcidin expression and promote macrophage iron retention and systemic hypoferremia [48], that complement activation and terminal pathway components are elevated in severe COVID-19, and that TFRC-mediated iron uptake is essential for lymphocyte activation and immune competence [49]. In addition, excessive iron uptake has been linked to impaired T-cell metabolism and inflammatory dysfunction in systemic autoimmune disease, consistent with a broader role for iron handling in immune regulation [50]. Nevertheless, direct experimental evidence connecting complement activation, TFRC regulation, iron redistribution, and B-cell functional remodeling in COVID-19 remains limited. Future studies should test whether complement activation products such as C3a/C5a, terminal complement complex formation, or complement-driven inflammatory signaling influence iron-regulatory pathways. They should also determine whether iron modulation can attenuate complement-associated tissue injury or immune dysfunction.

While our primary analysis focused on acute severe COVID-19, the persistence of complement–coagulation dysregulation beyond the acute phase has implications for Long COVID pathogenesis. A recent longitudinal study found that Long COVID patients show sustained complement activation and elevated serum vWF and F11 months after infection [34]. We hypothesize that the iron–complement network model identified in acute severe disease may similarly operate in prolonged thromboinflammatory states: persistent C9 elevation could drive chronic endothelial dysfunction, while ongoing iron redistribution (via TFRC dysregulation) may sustain immune metabolic exhaustion. However, this extrapolation is speculative, as our study did not include post-acute follow-up data. Testing whether TFRC and C9 remain elevated in Long COVID, and whether they correlate with specific symptom clusters (e.g., fatigue, cognitive impairment), represents an important extension of our work.

While our study offers valuable insights, limitations exist. First, the analyzed datasets were modest in size and represented different biological compartments, including serum, whole blood, and postmortem lung tissue. Although these datasets were analyzed separately and integrated at the level of pathway convergence, network features, immune-cell inference, and candidate molecule prioritization, cross-platform and cross-compartment heterogeneity may still influence interpretation. Second, the cross-sectional design precludes temporal inference regarding whether complement–coagulation network rewiring precedes immune-cell remodeling or occurs as a consequence of severe inflammation. Third, WGCNA and DiNA identify statistical associations and changes in correlation-derived network topology, but they do not establish regulatory relationships, direct protein–protein interactions, or causal mechanisms. Fourth, CIBERSORTx infers cell proportions from bulk transcriptomic profiles, and the estimates may be affected by tissue inflammation, altered cell states, RNA quality, and reference signature mismatch, particularly in postmortem lung tissues. Fifth, the study lacks experimental perturbation or targeted validation, such as TFRC knockdown, iron chelation, complement inhibition, ELISA/qPCR validation, or direct measurement of ferritin, transferrin saturation, C3a/C5a, lipid peroxidation, and GPX4. These limitations indicate that the proposed iron–complement network relationship should be considered a computationally derived hypothesis requiring further validation.

Future research should prioritize longitudinal and experimental studies to validate and extend these findings. Longitudinal cohorts are needed to distinguish acute thromboinflammatory and immune–metabolic alterations from persistent post-acute changes. Independent clinical cohorts should be used to evaluate TFRC, C9, and LBP as candidate molecular indicators using standardized assays and clinically relevant outcomes. Finally, perturbation-based studies, including complement inhibition, TFRC modulation, and iron metabolism intervention, will be necessary to test whether the coordinated network relationships identified here reflect causal biological interactions. Such studies would provide a more comprehensive understanding of severe COVID-19 pathophysiology and clarify the translational relevance of the candidate molecules prioritized by this integrative network analysis.

## Figures and Tables

**Table 1:** List of the identified hub proteins in the turquoise module.

Uniprot ID	Gene Symbol	Description	kWithin
P02748	C9	complement C9	42.98117079
P21926	CD9	CD9 molecule	40.82718145
P02763	ORM1	orosomucoid 1	39.57013516
P01011	SERPINA3	serpin family A member 3	35.92033201
P13671	C6	complement C6	35.57115683
P05155	SERPING1	serpin family G member 1	34.36167749
P04003	C4BPA	complement component 4 binding protein alpha	34.03476212
P18428	LBP	lipopolysaccharide binding protein	33.86072867
P04275	VWF	von Willebrand factor	33.34210331
P02768	ALB	albumin	32.23933855
P06681	C2	complement C2	32.01037133
Q14520	HABP2	hyaluronan binding protein 2	31.56126989
Q96PD5	PGLYRP2	peptidoglycan recognition protein 2	30.01011555
Q5SYB0	FRMPD1	FERM and PDZ domain containing 1	29.90994746
P00738	HP	haptoglobin	29.42244338
P22792	CPN2	carboxypeptidase N subunit 2	28.07670488
P04196	HRG	histidine rich glycoprotein	26.46408776
P03951	F11	coagulation factor XI	26.42462227
P06727	APOA4	apolipoprotein A4	24.61680441
Q9UHG3	PCYOX1	prenylcysteine oxidase 1	22.76211877

## Data Availability

Data related to this study are available within the main text and the Supplementary Files. All other data are available from the corresponding author upon reasonable request.

## Supplementary Materials

The following supporting information can be downloaded at: https://www.mdpi.com/article/10.3390/cimb48050536/s1.
