# Supplementary material for: An Iron–Complement Network Model of Thromboinflammation and Humoral Immune Remodeling in Severe COVID-19
Source: Curr Issues Mol Biol. 2026 May 21;48(5):536. doi: 10.3390/cimb48050536 (PMC13206193; doi:10.3390/cimb48050536)
Supplement: Supplementary file 1 [file cimb-48-00536-s001.zip › cimb-4266250-supplementary.pdf]

Supplementary material

Table S1

Table S1. The module proteins significantly associated with severe COVID-19.

Uniprot ID	Gene Symbol	Description
P04114	APOB	apolipoprotein B
P01024	C3	complement C3
P02768	ALB	albumin
P01023	A2M	alpha-2-macroglobulin
P02751	FN1	fibronectin 1
P0C0L5	C4B	complement C4B (Chido blood group)
P0C0L4	C4A	complement C4A (Rodgers blood group)
P02787	TF	transferrin
P00450	CP	ceruloplasmin
P08603	CFH	complement factor H
P01031	C5	complement C5
P04275	VWF	von Willebrand factor
P01009	SERPINA1	serpin family A member 1
P02774	GC	GC vitamin D binding protein
P00738	HP	haptoglobin
Q14624	ITIH4	inter-alpha-trypsin inhibitor heavy chain 4
P02647	APOA1	apolipoprotein A1
P10643	C7	complement C7
P01008	SERPINC1	serpin family C member 1
P00751	CFB	complement factor B
P13671	C6	complement C6
P01042	KNG1	kininogen 1
P19827	ITIH1	inter-alpha-trypsin inhibitor heavy chain 1
P06396	GSN	gelsolin
P07996	THBS1	thrombospondin 1
P09871	C1S	complement C1s
P01011	SERPINA3	serpin family A member 3
P12259	F5	coagulation factor V
P06727	APOA4	apolipoprotein A4
P04003	C4BPA	complement component 4 binding protein alpha
P19823	ITIH2	inter-alpha-trypsin inhibitor heavy chain 2
P20742	PZP	PZP alpha-2-macroglobulin like
P02671	FGA	fibrinogen alpha chain
P00736	C1R	complement C1r
Q06033	ITIH3	inter-alpha-trypsin inhibitor heavy chain 3
P02749	APOH	apolipoprotein H

Uniprot ID	Gene Symbol	Description
P06681	C2	complement C2
P43652	AFM	afamin
P05156	CFI	complement factor I
P05155	SERPING1	serpin family G member 1
P07225	PROS1	protein S
P02675	FGB	fibrinogen beta chain
P07357	C8A	complement C8 alpha chain
P02748	C9	complement C9
P07358	C8B	complement C8 beta chain
P04217	A1BG	alpha-1-B glycoprotein
O43866	CD5L	CD5 molecule like
P25311	AZGP1	alpha-2-glycoprotein 1, zinc-binding
P04196	HRG	histidine rich glycoprotein
P29622	SERPINA4	serpin family A member 4
P02679	FGG	fibrinogen gamma chain
P05543	SERPINA7	serpin family A member 7
P80108	GPLD1	glycosylphosphatidylinositol specific phospholipase D1
P02765	AHSG	alpha 2-HS glycoprotein
P02763	ORM1	orosomucoid 1
P27169	PON1	paraoxonase 1
P00742	F10	coagulation factor X
P13796	LCP1	lymphocyte cytosolic protein 1
P08697	SERPINF2	serpin family F member 2
Q14520	HABP2	hyaluronan binding protein 2
Q16610	ECM1	extracellular matrix protein 1
P19652	ORM2	orosomucoid 2
P04004	VTN	vitronectin
Q08380	LGALS3BP	galectin 3 binding protein
Q92954	PRG4	proteoglycan 4
P00748	F12	coagulation factor XII
P02652	APOA2	apolipoprotein A2
P01019	AGT	angiotensinogen
Q15582	TGFBI	transforming growth factor beta induced
P00740	F9	coagulation factor IX
Q9BXR6	CFHR5	complement factor H related 5
Q03591	CFHR1	complement factor H related 1
O14791	APOL1	apolipoprotein L1
P02753	RBP4	retinol binding protein 4
Q96PD5	PGLYRP2	peptidoglycan recognition protein 2
P03951	F11	coagulation factor XI
P08185	SERPINA6	serpin family A member 6
P07360	C8G	complement C8 gamma chain
Q04756	HGFAC	HGF activator

Uniprot ID	Gene Symbol	Description
P05090	APOD	apolipoprotein D
P02750	LRG1	leucine rich alpha-2-glycoprotein 1
P20851	C4BPB	complement component 4 binding protein beta
P15169	CPN1	carboxypeptidase N subunit 1
P22792	CPN2	carboxypeptidase N subunit 2
P51884	LUM	lumican
Q02985	CFHR3	complement factor H related 3
P0DJI8	SAA1	serum amyloid A1
O75636	FCN3	ficolin 3
P49747	COMP	cartilage oligomeric matrix protein
P36980	CFHR2	complement factor H related 2
P18428	LBP	lipopolysaccharide binding protein
Q9NZP8	C1RL	complement C1r subcomponent like
P06276	BCHE	butyrylcholinesterase
P13645	KRT10	keratin 10
P00746	CFD	complement factor D
Q12805	EFEMP1	EGF containing fibulin extracellular matrix protein 1
P19320	VCAM1	vascular cell adhesion molecule 1
Q9UHG3	PCYOX1	prenylcysteine oxidase 1
P17936	IGFBP3	insulin like growth factor binding protein 3
P02743	APCS	amyloid P component, serum
P35908	KRT2	keratin 2
Q9UGM5	FETUB	fetuin B
P05452	CLEC3B	C-type lectin domain family 3 member B
P22105	TNXB	tenascin XB
O00187	MASP2	MBL associated serine protease 2
Q14766	LTBP1	latent transforming growth factor beta binding protein 1
P27918	CFP	complement factor properdin
Q9NQ79	CRTAC1	cartilage acidic protein 1
P33151	CDH5	cadherin 5
Q9UK55	SERPINA10	serpin family A member 10
P98160	HSPG2	heparan sulfate proteoglycan 2
Q92496	CFHR4	complement factor H related 4
Q9Y490	TLN1	talin 1
P0DJI9	SAA2	serum amyloid A2
Q15485	FCN2	ficolin 2
P02656	APOC3	apolipoprotein C3
P08571	CD14	CD14 molecule
P02654	APOC1	apolipoprotein C1
O00391	QSOX1	quiescin sulfhydryl oxidase 1
Q92820	GGH	gamma-glutamyl hydrolase
P02655	APOC2	apolipoprotein C2
Q13201	MMRN1	multimerin 1

Uniprot ID	Gene Symbol	Description
P33908	MAN1A1	mannosidase alpha class 1A member 1
P40197	GP5	glycoprotein V platelet
Q9HDC9	APMAP	adipocyte plasma membrane associated protein
O95445	APOM	apolipoprotein M
P05062	ALDOB	aldolase, fructose-bisphosphate B
P04180	LCAT	lecithin-cholesterol acyltransferase
P09486	SPARC	secreted protein acidic and cysteine rich
P35542	SAA4	serum amyloid A4, constitutive
P07737	PFN1	profilin 1
P14543	NID1	nidogen 1
Q6UXB8	PI16	peptidase inhibitor 16
P02775	PPBP	pro-platelet basic protein
P02745	C1QA	complement C1q A chain
P06702	S100A9	S100 calcium binding protein A9
Q15166	PON3	paraoxonase 3
P02776	PF4	platelet factor 4
Q15113	PCOLCE	procollagen C-endopeptidase enhancer
P07195	LDHB	lactate dehydrogenase B
P01034	CST3	cystatin C
P21333	FLNA	filamin A
P04406	GAPDH	glyceraldehyde-3-phosphate dehydrogenase
A0A0B4J1V6	IGHV3-73	immunoglobulin heavy variable 3-73
P02741	CRP	C-reactive protein
P49908	SELENOP	selenoprotein P
P05534	HLA-A	major histocompatibility complex, class I, A
P08253	MMP2	matrix metallopeptidase 2
A0A0C4DH73	IGKV1-12	immunoglobulin kappa variable 1-12
P07339	CTSD	cathepsin D
P10720	PF4V1	platelet factor 4 variant 1
A0A0B4J2H0	IGHV1-69D	immunoglobulin heavy variable 1-69D
P55058	PLTP	phospholipid transfer protein
P07359	GP1BA	glycoprotein Ib platelet subunit alpha
Q12913	PTPRJ	protein tyrosine phosphatase receptor type J
P13647	KRT5	keratin 5
P14625	HSP90B1	heat shock protein 90 beta family member 1
Q9Y5Y7	LYVE1	lymphatic vessel endothelial hyaluronan receptor 1
P06733	ENO1	enolase 1
P55056	APOC4	apolipoprotein C4
Q16706	MAN2A1	mannosidase alpha class 2A member 1
P07988	SFTPB	surfactant protein B
Q86UD1	OAF	out at first homolog
P54108	CRISP3	cysteine rich secretory protein 3
P43121	MCAM	melanoma cell adhesion molecule

Uniprot ID	Gene Symbol	Description
Q9UNW1	MINPP1	multiple inositol-polyphosphate phosphatase 1
Q08830	FGL1	fibrinogen like 1
Q6UWP8	SBSN	suprabasin
P62937	PPIA	peptidylprolyl isomerase A
Q8NBP7	PCSK9	proprotein convertase subtilisin/kexin type 9
P35916	FLT4	fms related receptor tyrosine kinase 4
P05362	ICAM1	intercellular adhesion molecule 1
P26038	MSN	moesin
P29401	TKT	transketolase
O14786	NRP1	neuropilin 1
P18065	IGFBP2	insulin like growth factor binding protein 2
P00338	LDHA	lactate dehydrogenase A
Q9Y5C1	ANGPTL3	angiopoietin like 3
P22692	IGFBP4	insulin like growth factor binding protein 4
Q9NPH3	IL1RAP	interleukin 1 receptor accessory protein
P08637	FCGR3A	Fc gamma receptor IIIa
P23528	CFL1	cofilin 1
O95479	H6PD	hexose-6-phosphate dehydrogenase/glucose 1-dehydrogenase
P24821	TNC	tenascin C
P37802	TAGLN2	transgelin 2
P67936	TPM4	tropomyosin 4
Q86YW5	TREML1	triggering receptor expressed on myeloid cells like 1
P61769	B2M	beta-2-microglobulin
P09960	LTA4H	leukotriene A4 hydrolase
P55103	INHBC	inhibin subunit beta C
P07998	RNASE1	ribonuclease A family member 1, pancreatic
Q6YHK3	CD109	CD109 molecule
P24593	IGFBP5	insulin like growth factor binding protein 5
P08294	SOD3	superoxide dismutase 3
P11597	CETP	cholesteryl ester transfer protein
Q6Q788	APOA5	apolipoprotein A5
P62805	H4C1	H4 clustered histone 1
P01344	IGF2	insulin like growth factor 2
P61204	ARF3	ADP ribosylation factor 3
Q16853	AOC3	amine oxidase copper containing 3
P07942	LAMB1	laminin subunit beta 1
P81605	DCD	dermcidin
Q9H8L6	MMRN2	multimerin 2
P01137	TGFB1	transforming growth factor beta 1
P30740	SERPINB1	serpin family B member 1
P23284	PPIB	peptidylprolyl isomerase B
Q5QNW6	H2BC18	H2B clustered histone 18
Q13822	ENPP2	ectonucleotide pyrophosphatase/phosphodiesterase 2

Uniprot ID	Gene Symbol	Description
P27797	CALR	calreticulin
Q8NBJ4	GOLM1	golgi membrane protein 1
P16930	FAH	fumarylacetoacetate hydrolase
O00151	PDLIM1	PDZ and LIM domain 1
O75144	ICOSLG	inducible T cell costimulator ligand
P13727	PRG2	proteoglycan 2, pro eosinophil major basic protein
P40189	IL6ST	interleukin 6 cytokine family signal transducer
P05121	SERPINE1	serpin family E member 1
P15151	PVR	PVR cell adhesion molecule
P08246	ELANE	elastase, neutrophil expressed
P02792	FTL	ferritin light chain
Q8WWZ8	OIT3	oncoprotein induced transcript 3
P54802	NAGLU	N-acetyl-alpha-glucosaminidase
P80511	S100A12	S100 calcium binding protein A12
P08514	ITGA2B	integrin subunit alpha 2b
P01705	IGLV2-23	immunoglobulin lambda variable 2-23
P28827	PTPRM	protein tyrosine phosphatase receptor type M
Q9NPY3	CD93	CD93 molecule
P17813	ENG	endoglin
A0A0C4DH39	IGHV1-58	immunoglobulin heavy variable 1-58
Q86TH1	ADAMTSL2	ADAMTS like 2
Q12794	HYAL1	hyaluronidase 1
P59665	DEFA1	defensin alpha 1
Q9UEW3	MARCO	macrophage receptor with collagenous structure
Q8WUA8	TSKU	tsukushi, small leucine rich proteoglycan
P05019	IGF1	insulin like growth factor 1
O75874	IDH1	isocitrate dehydrogenase (NADP(+)) 1
Q8IXL6	FAM20C	FAM20C golgi associated secretory pathway kinase
P27487	DPP4	dipeptidyl peptidase 4
P31995	FCGR2C	Fc gamma receptor IIc (gene/pseudogene)
P13987	CD59	CD59 molecule (CD59 blood group)
Q16777	H2AC20	H2A clustered histone 20
P28799	GRN	granulin precursor
Q12907	LMAN2	lectin, mannose binding 2
Q9UNN8	PROCR	protein C receptor
Q92859	NEO1	neogenin 1
P84243	H3-3A	H3.3 histone A
P00390	GSR	glutathione-disulfide reductase
P46531	NOTCH1	notch receptor 1
Q8IZF2	ADGRF5	adhesion G protein-coupled receptor F5
Q99453	PHOX2B	paired like homeobox 2B
Q15942	ZYX	zyxin
Q5SYB0	FRMPD1	FERM and PDZ domain containing 1

Uniprot ID	Gene Symbol	Description
P21926	CD9	CD9 molecule
P46109	CRKL	CRK like proto-oncogene, adaptor protein
Q9Y274	ST3GAL6	ST3 beta-galactoside alpha-2,3-sialyltransferase 6
Q5JXB2	UBE2NL	ubiquitin conjugating enzyme E2 N like (gene/pseudogene)
Q9HCN6	GP6	glycoprotein VI platelet
Q14697	GANAB	glucosidase II alpha subunit
Q8N3J3	HROB	homologous recombination factor with OB-fold
Q92887	ABCC2	ATP binding cassette subfamily C member 2
P28066	PSMA5	proteasome 20S subunit alpha 5
P30531	SLC6A1	solute carrier family 6 member 1
Q6DHV5	CC2D2B	coiled-coil and C2 domain containing 2B
Q9UBX5	FBLN5	fibulin 5

Table S2

Table S2. Top 20 entries for gene pathway enrichment analysis.

GO	Category	Description	Count	%	Log10(P)	Log10(q)
hsa04610	KEGG Pathway	Complement and coagulation cascades	46	17.49	-72.29	-68.01
R-HSA-114608	Reactome Gene Sets	Platelet degranulation	49	18.63	-67.19	-63.22
R-HSA-381426	Reactome Gene Sets	Regulation of Insulin-like Growth Factor (IGF) transport and uptake by Insulin-like Growth Factor Binding Proteins (IGFBPs)	37	14.07	-45.79	-42.41
WP5115	WikiPathways	Network map of SARS-CoV-2 signaling pathway	40	15.21	-40.09	-36.93
GO:0051346	GO Biological Processes	negative regulation of hydrolase activity	40	15.21	-30.88	-27.95
GO:0006954	GO Biological Processes	inflammatory response	42	15.97	-27.88	-25.09
R-HSA-6798695	Reactome Gene Sets	Neutrophil degranulation	36	13.69	-22.3	-19.7
GO:0006957	GO Biological Processes	complement activation, alternative pathway	12	4.56	-22.18	-19.59
R-HSA-1474244	Reactome Gene Sets	Extracellular matrix organization	29	11.03	-21	-18.44
GO:0034368	GO Biological Processes	protein-lipid complex remodeling	13	4.94	-19.13	-16.63
R-HSA-140837	Reactome Gene Sets	Intrinsic Pathway of Fibrin Clot Formation	12	4.56	-18.74	-16.26
GO:0030155	GO Biological Processes	regulation of cell adhesion	39	14.83	-18.31	-15.86
GO:0001775	GO Biological Processes	cell activation	34	12.93	-16.5	-14.09
GO:0009617	GO Biological Processes	response to bacterium	36	13.69	-16.43	-14.03
WP15	WikiPathways	Selenium micronutrient network	16	6.08	-15.92	-13.54

GO	Category	Description	Count	%	Log10(P)	Log10(q)
GO:0051702	GO Biological Processes	biological process involved in interaction with symbiont	15	5.7	-14.1	-11.76
GO:0031638	GO Biological Processes	zymogen activation	12	4.56	-13.88	-11.55
GO:0060627	GO Biological Processes	regulation of vesicle-mediated transport	28	10.65	-13.52	-11.22
GO:2000147	GO Biological Processes	positive regulation of cell motility	29	11.03	-13.29	-11
R-HSA-2173782	Reactome Gene Sets	Binding and Uptake of Ligands by Scavenger Receptors	11	4.18	-13.23	-10.94

Table S3

Table S3. Hub proteins in the non-conservative module.

Uniprot ID	Gene Symbol	Description	K1	K2	DiffK
P02750	LRG1	leucine rich alpha-2-glycoprotein 1	0.150109	1	0.849891
P0DOX7	NA	Immunoglobulin kappa light chain	1	0.163905	0.836095
P02748	C9	complement C9	0.078241	0.852911	0.77467
P01834	IGKC	immunoglobulin kappa constant	0.980397	0.235778	0.744619
Q06830	PRDX1	peroxiredoxin 1	0.117771	0.850442	0.73267
P02786	TFRC	transferrin receptor	0.085981	0.803695	0.717715
Q06033	ITIH3	inter-alpha-trypsin inhibitor heavy chain 3	0.123324	0.801225	0.677901
P01009	SERPINA1	serpin family A member 1	0.198155	0.84669	0.648536
Q96K62	ZBTB45	zinc finger and BTB domain containing 45	0.736248	0.092912	0.643336
P18428	LBP	lipopolysaccharide binding protein	0.106811	0.743752	0.636941
P16035	TIMP2	TIMP metallopeptidase inhibitor 2	0.730852	0.119608	0.611244
A0A075B6K0	IGLV3-16	immunoglobulin lambda variable 3-16	0.664867	0.074454	0.590413
P33908	MAN1A1	mannosidase alpha class 1A member 1	0.238948	0.827389	0.588441
P0DOX3	NA	Immunoglobulin delta heavy chain	0.148519	0.736261	0.587743
A0A0C4DH55	IGKV3D-7	immunoglobulin kappa variable 3D-7	0.804054	0.222583	0.581472
P06312	IGKV4-1	immunoglobulin kappa variable 4-1	0.838446	0.261267	0.57718
Q9NZP8	C1RL	complement C1r subcomponent like	0.140189	0.717144	0.576955
P0DP03	IGHV3-30-5	immunoglobulin heavy variable 3-30-5	0.806345	0.234524	0.571821
P14174	MIF	macrophage migration inhibitory factor	0.155628	0.721296	0.565668
Q08380	LGALS3BP	galectin 3 binding protein	0.117282	0.682499	0.565218

Figure S1

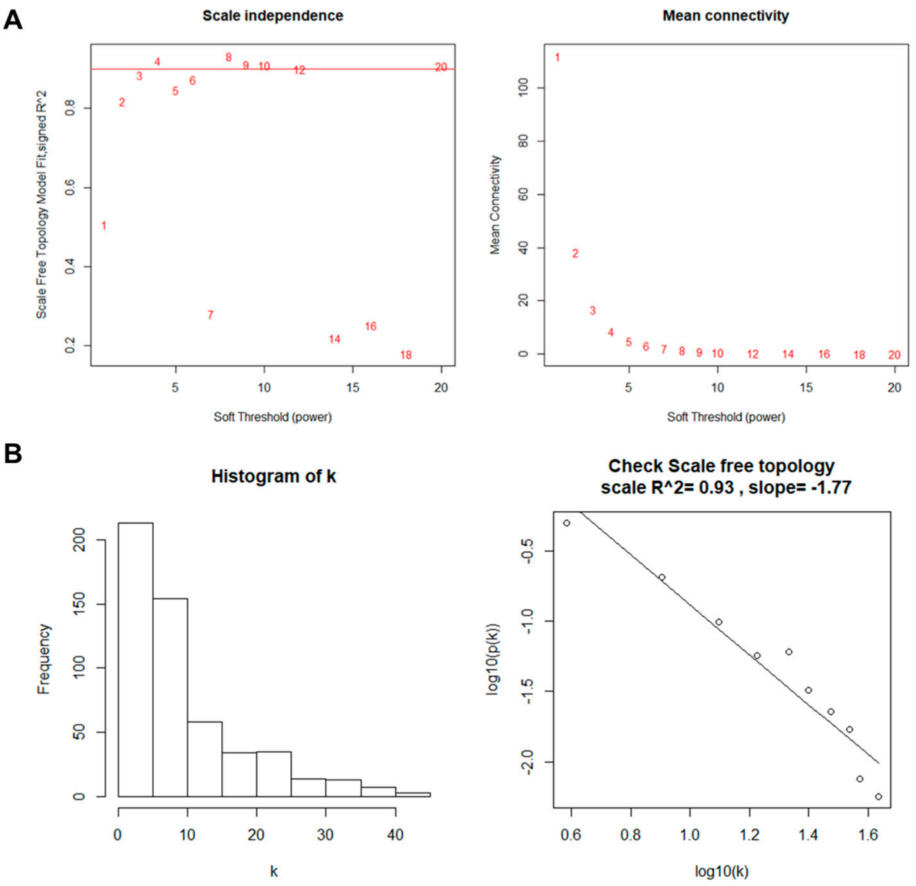

Figure S1. WGCNA soft-thresholding power selection. (A) Scale-free topology fit index and mean connectivity across candidate powers. $\beta = 3$ was selected because it was the first value reaching a scale-free topology fit index of 0.85 while maintaining network connectivity. **(B)** Scale-free topology diagnostic at $\beta = 3$, showing an approximately linear log–log connectivity distribution ($R^2 = 0.93$, slope $= -1.77$).

Figure S2

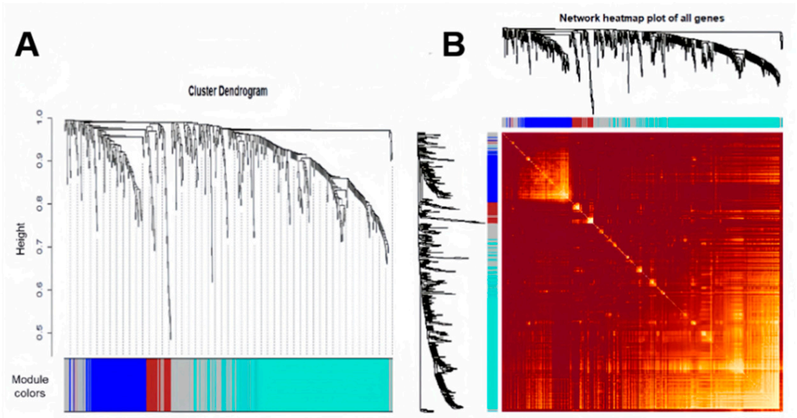

Figure S2. Module and network plots. (A) Gene dendrogram obtained by hierarchical clustering. The color row underneath the dendrogram shows the module assignment. **(B)** Network heatmap plot. Heatmap plot of topological overlap matrix dissimilarity in the gene network. Light color denotes high overlap and darker red color denotes low topological overlap. The gene dendrogram and color-coded module are displayed along the left and top.

Figure S3

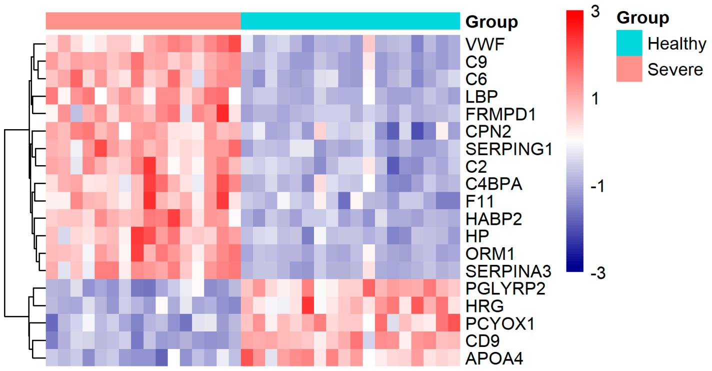

Figure S3. Expression of hub proteins in COVID-19 severe patients and healthy controls.

Figure S4

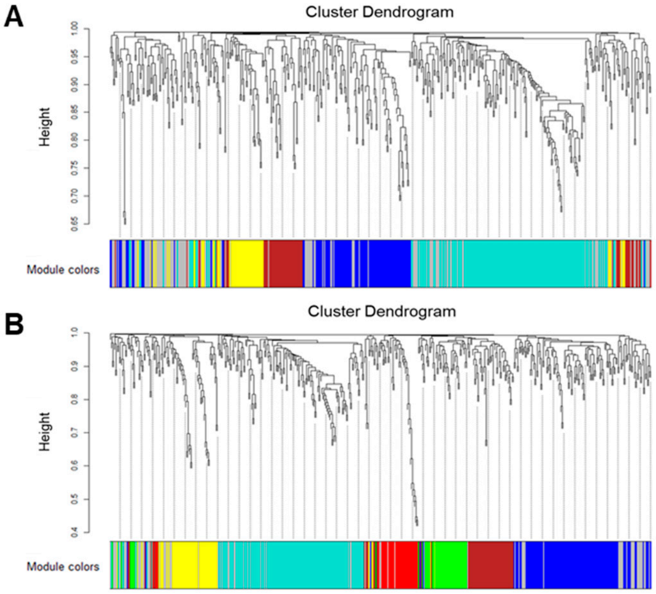

Figure S4. Co-expression modules identified and characterized by WGCNA. (A) Clustering dendrograms of healthy controls. **(B)** Clustering dendrograms of severe COVID-19 patients.

Figure S5

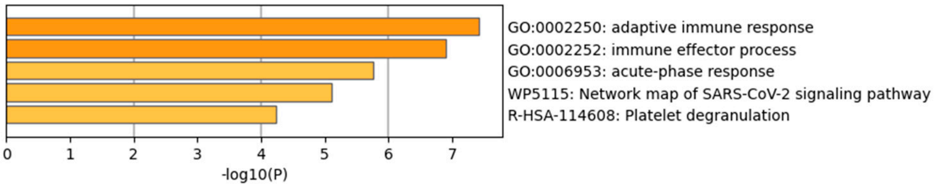

Figure S5. Bar graph of enriched terms across hub proteins, colored by p-values.

Figure S6

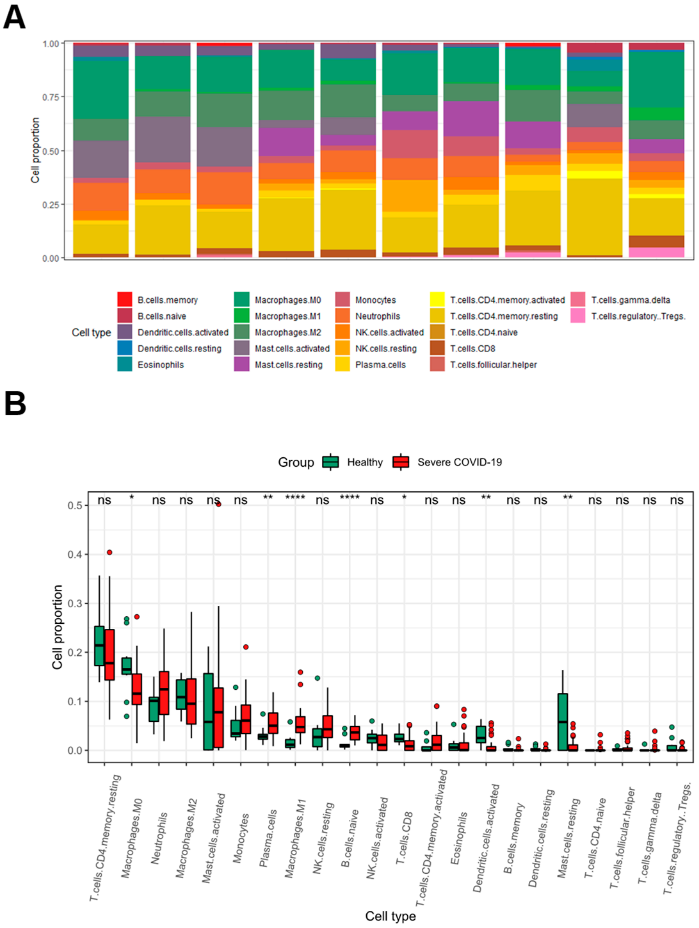

Figure S6. Immune cell subset proportions and distribution in healthy and severe COVID-19 lung tissue. (A) Proportion of 22 immune cell subsets in healthy lung tissue. **(B)** Distribution of different immune cell subsets between severe COVID-19 and healthy controls (p-values from Wilcoxon test). ns, not significant; $*p < 0.05$; $**p < 0.01$; $***p < 0.001$; $****p < 0.0001$.
